# Supplementary material for: HIVprotI: an integrated web based platform for prediction and design of HIV proteins inhibitors
Source: J Cheminform. 2018 Mar 9;10:12. doi: 10.1186/s13321-018-0266-y (PMC5845081; doi:10.1186/s13321-018-0266-y)
Supplement: Supplementary file 2 — Additional file 2. Source code of HIVProtI web server. [file 13321_2018_266_MOESM2_ESM.zip › HIVprotI_Source-code/hivprotI/weka-3-6-6/doc/weka/core/Trie.html]

Trie


---


|  |  |  |
| --- | --- | --- |
| **Overview** \| **Package** \| **Class** \| **Tree** \| **Deprecated** \| **Index** \| **Help** \| **Weka's home** | |  |
| **PREV CLASS**   **NEXT CLASS** | **FRAMES**    **NO FRAMES**     **All Classes** |  |
| SUMMARY: NESTED \| FIELD \| CONSTR \| METHOD | DETAIL: FIELD \| CONSTR \| METHOD |  |


---


## weka.core Class Trie

```
java.lang.Object
  weka.core.Trie
```

**All Implemented Interfaces:**: java.io.Serializable, java.lang.Cloneable, java.lang.Iterable<java.lang.String>, java.util.Collection<java.lang.String>, RevisionHandler

---

``` public class Trie extends java.lang.Object implements java.io.Serializable, java.lang.Cloneable, java.util.Collection<java.lang.String>, RevisionHandler ```

A class representing a Trie data structure for strings.
See also Trie
on WikiPedia.

**Version:**
:   $Revision: 1.2 $

**Author:**
:   fracpete (fracpete at waikato dot ac dot nz)

**See Also:**: Serialized Form

---

| **Nested Class Summary** | |
| --- | --- |
| `static class` | `Trie.TrieIterator`             Represents an iterator over a trie |
| `static class` | `Trie.TrieNode`             Represents a node in the trie. |


**Constructor Summary**

`Trie()`
  
          initializes the data structure


| **Method Summary** | |
| --- | --- |
| `boolean` | `add(java.lang.String o)`             Ensures that this collection contains the specified element. |
| `boolean` | `addAll(java.util.Collection<? extends java.lang.String> c)`             Adds all of the elements in the specified collection to this collection |
| `void` | `clear()`             Removes all of the elements from this collection |
| `java.lang.Object` | `clone()`             returns a deep copy of itself |
| `boolean` | `contains(java.lang.Object o)`             Returns true if this collection contains the specified element. |
| `boolean` | `containsAll(java.util.Collection<?> c)`             Returns true if this collection contains all of the elements in the specified collection. |
| `boolean` | `containsPrefix(java.lang.String prefix)`             checks whether the given prefix is stored in the trie |
| `boolean` | `equals(java.lang.Object o)`             Compares the specified object with this collection for equality. |
| `java.lang.String` | `getCommonPrefix()`             returns the common prefix for all the nodes |
| `java.lang.String` | `getRevision()`             Returns the revision string. |
| `Trie.TrieNode` | `getRoot()`             returns the root node of the trie |
| `java.util.Vector<java.lang.String>` | `getWithPrefix(java.lang.String prefix)`             returns all stored strings that match the given prefix |
| `int` | `hashCode()`             Returns the hash code value for this collection. |
| `boolean` | `isEmpty()`             Returns true if this collection contains no elements. |
| `java.util.Iterator<java.lang.String>` | `iterator()`             Returns an iterator over the elements in this collection. |
| `static void` | `main(java.lang.String[] args)`             Only for testing (prints the built Trie). |
| `boolean` | `remove(java.lang.Object o)`             Removes a single instance of the specified element from this collection, if it is present. |
| `boolean` | `removeAll(java.util.Collection<?> c)`             Removes all this collection's elements that are also contained in the specified collection |
| `boolean` | `retainAll(java.util.Collection<?> c)`             Retains only the elements in this collection that are contained in the specified collection |
| `int` | `size()`             Returns the number of elements in this collection. |
| `java.lang.Object[]` | `toArray()`             Returns an array containing all of the elements in this collection. |
| `<T> T[]` | `toArray(T[] a)`             Returns an array containing all of the elements in this collection; the runtime type of the returned array is that of the specified array. |
| `java.lang.String` | `toString()`             returns the trie in string representation |

**Methods inherited from class java.lang.Object**

`getClass, notify, notifyAll, wait, wait, wait`

**Constructor Detail**

### Trie

```
public Trie()
```

:   initializes the data structure


**Method Detail**

### add

```
public boolean add(java.lang.String o)
```

:   Ensures that this collection contains the specified element.

    :   **Specified by:**: `add` in interface `java.util.Collection<java.lang.String>`
    :   **Parameters:**: `o` - the string to add **Returns:**: true if the structure changed

---


### addAll

```
public boolean addAll(java.util.Collection<? extends java.lang.String> c)
```

:   Adds all of the elements in the specified collection to this collection

    :   **Specified by:**: `addAll` in interface `java.util.Collection<java.lang.String>`
    :   **Parameters:**: `c` - the collection to add

---


### clear

```
public void clear()
```

:   Removes all of the elements from this collection

    :   **Specified by:**: `clear` in interface `java.util.Collection<java.lang.String>`

---


### clone

```
public java.lang.Object clone()
```

:   returns a deep copy of itself

    :   **Overrides:**: `clone` in class `java.lang.Object`
    :   **Returns:**: a copy of itself

---


### contains

```
public boolean contains(java.lang.Object o)
```

:   Returns true if this collection contains the specified element.

    :   **Specified by:**: `contains` in interface `java.util.Collection<java.lang.String>`
    :   **Parameters:**: `o` - the object to check for in trie **Returns:**: true if found

---


### containsAll

```
public boolean containsAll(java.util.Collection<?> c)
```

:   Returns true if this collection contains all of the elements in the
    specified collection.

    :   **Specified by:**: `containsAll` in interface `java.util.Collection<java.lang.String>`
    :   **Parameters:**: `c` - the collection to look for in the trie **Returns:**: true if all elements were found

---


### containsPrefix

```
public boolean containsPrefix(java.lang.String prefix)
```

:   checks whether the given prefix is stored in the trie

    :   **Parameters:**: `prefix` - the prefix to check **Returns:**: true if the prefix is part of the trie

---


### equals

```
public boolean equals(java.lang.Object o)
```

:   Compares the specified object with this collection for equality.

    :   **Specified by:**: `equals` in interface `java.util.Collection<java.lang.String>` **Overrides:**: `equals` in class `java.lang.Object`
    :   **Parameters:**: `o` - the object to check for equality

---


### getCommonPrefix

```
public java.lang.String getCommonPrefix()
```

:   returns the common prefix for all the nodes

    :   **Returns:**: the result of the search

---


### getRoot

```
public Trie.TrieNode getRoot()
```

:   returns the root node of the trie

    :   **Returns:**: the root node

---


### getWithPrefix

```
public java.util.Vector<java.lang.String> getWithPrefix(java.lang.String prefix)
```

:   returns all stored strings that match the given prefix

    :   **Parameters:**: `prefix` - the prefix that all strings must have **Returns:**: all strings that match the prefix

---


### hashCode

```
public int hashCode()
```

:   Returns the hash code value for this collection.

    :   **Specified by:**: `hashCode` in interface `java.util.Collection<java.lang.String>` **Overrides:**: `hashCode` in class `java.lang.Object`
    :   **Returns:**: the hash code

---


### isEmpty

```
public boolean isEmpty()
```

:   Returns true if this collection contains no elements.

    :   **Specified by:**: `isEmpty` in interface `java.util.Collection<java.lang.String>`
    :   **Returns:**: true if empty

---


### iterator

```
public java.util.Iterator<java.lang.String> iterator()
```

:   Returns an iterator over the elements in this collection.

    :   **Specified by:**: `iterator` in interface `java.lang.Iterable<java.lang.String>` **Specified by:**: `iterator` in interface `java.util.Collection<java.lang.String>`
    :   **Returns:**: returns an iterator over all the stored strings

---


### remove

```
public boolean remove(java.lang.Object o)
```

:   Removes a single instance of the specified element from this collection,
    if it is present.

    :   **Specified by:**: `remove` in interface `java.util.Collection<java.lang.String>`
    :   **Parameters:**: `o` - the object to remove **Returns:**: true if this collection changed as a result of the call

---


### removeAll

```
public boolean removeAll(java.util.Collection<?> c)
```

:   Removes all this collection's elements that are also contained in the
    specified collection

    :   **Specified by:**: `removeAll` in interface `java.util.Collection<java.lang.String>`
    :   **Parameters:**: `c` - the collection to remove **Returns:**: true if the collection changed

---


### retainAll

```
public boolean retainAll(java.util.Collection<?> c)
```

:   Retains only the elements in this collection that are contained in
    the specified collection

    :   **Specified by:**: `retainAll` in interface `java.util.Collection<java.lang.String>`
    :   **Parameters:**: `c` - the collection to use as reference **Returns:**: true if this collection changed as a result of the call

---


### size

```
public int size()
```

:   Returns the number of elements in this collection.

    :   **Specified by:**: `size` in interface `java.util.Collection<java.lang.String>`
    :   **Returns:**: the number of nodes in the tree

---


### toArray

```
public java.lang.Object[] toArray()
```

:   Returns an array containing all of the elements in this collection.

    :   **Specified by:**: `toArray` in interface `java.util.Collection<java.lang.String>`
    :   **Returns:**: the stored strings as array

---


### toArray

```
public <T> T[] toArray(T[] a)
```

:   Returns an array containing all of the elements in this collection; the
    runtime type of the returned array is that of the specified array.

    :   **Specified by:**: `toArray` in interface `java.util.Collection<java.lang.String>`
    :   **Parameters:**: `a` - the array into which the elements of this collection are to be stored **Returns:**: an array containing the elements of this collection

---


### toString

```
public java.lang.String toString()
```

:   returns the trie in string representation

    :   **Overrides:**: `toString` in class `java.lang.Object`
    :   **Returns:**: the trie as string

---


### getRevision

```
public java.lang.String getRevision()
```

:   Returns the revision string.

    :   **Specified by:**: `getRevision` in interface `RevisionHandler`
    :   **Returns:**: the revision

---


### main

```
public static void main(java.lang.String[] args)
```

:   Only for testing (prints the built Trie). Arguments are added to the Trie.
    If not arguments provided then a few default strings are uses for building.

    :   **Parameters:**: `args` - commandline arguments


---


|  |  |  |
| --- | --- | --- |
| **Overview** \| **Package** \| **Class** \| **Tree** \| **Deprecated** \| **Index** \| **Help** \| **Weka's home** | |  |
| **PREV CLASS**   **NEXT CLASS** | **FRAMES**    **NO FRAMES**     **All Classes** |  |
| SUMMARY: NESTED \| FIELD \| CONSTR \| METHOD | DETAIL: FIELD \| CONSTR \| METHOD |  |


---
